# Experiences of healthcare providers and caregivers regarding health system bottlenecks affecting child healthcare service delivery in a rural district: Empirical qualitative study

**DOI:** 10.1002/nop2.2228

**Published:** 2024-07-08

**Authors:** Felix Kwasi Nyande, Esmeralda Ricks, Margaret Williams, Sihaam Jardien‐Baboo

**Affiliations:** ^1^ Department of Nursing, School of Nursing and Midwifery University of Health and Allied Sciences Ho Ghana; ^2^ Department of Nursing Science, Faculty of Health Sciences Nelson Mandela University Gqeberha South Africa; ^3^ Faculty of Health Sciences Nelson Mandela University Gqeberha South Africa

**Keywords:** caregivers, child healthcare, health system bottlenecks, logistic constraints, shortage of medicines

## Abstract

**Aim:**

To explore the experiences of nurses and caregivers about the health system bottlenecks to the delivery of child healthcare services in a rural district in Ghana.

**Design:**

The study employed a qualitative approach using an exploratory, descriptive design.

**Methods:**

Collection of data was through semi‐structured, face‐to‐face interviews with 26 participants in the Nkwanta South Municipality, Ghana. Audio recordings of interviews were transcribed verbatim and analysed qualitatively. Inductive codes generated were organised into themes and sub‐themes.

**Results:**

The main health system bottlenecks that emerged were the poor state of in‐patient facilities, inadequate basic logistics and persistent shortage of essential medicines needed for child healthcare delivery.

**Conclusions:**

Health system bottlenecks have the tendency to affect the treatment and hospitalisation outcomes of sick children and eventually impact the state of child healthcare negatively. Concerted efforts by government and local authorities to remove these barriers will help to improve child health and child health outcomes.

**Public Contribution:**

A total of 26 participants comprising nurses and caregivers, agreed and participated in this study. Interviews with these participants were conducts either in the health facilities or in the communities where they live. Their responses contributed significantly to the content of this article.

## 1 INTRODUCTION

Children, particularly those under 5 years of age, constitute a vulnerable population which is at increased risk of numerous diseases leading to increased mortalities and morbidity (Berr et al., 2021; Lam et al., 2014; Pina et al., 2020; Sarfo et al., 2023; Tadele et al., 2022). The vulnerability of these children stems from their under‐developed organs and immune systems and their inability to fend for themselves. Globally, it is estimated that in 2019 alone, about 5.3 million children under 5 years of age died (Sharrow et al., 2022). Child mortalities and morbidities are the poorest in sub‐Saharan Africa (SSA), where most deaths in children under 5 years are a result of preventable or treatable causes (UN Inter‐agency Group for Child Mortality Estimation, 2021). According to the Ghana Demographic Health Survey (GDHS), under‐5 mortality rates in urban and rural Ghana stand at 37 and 42 deaths per 1000 live births, respectively (Ghana Statistical Service & ICF, 2023). Again, whereas the national under‐5 death rate stood at 40 deaths per live births, the Oti Region recorded 72 deaths per 1000 live births (Ghana Statistical Service & ICF, 2023).

Preventable and treatable childhood disease conditions, including malaria, pneumonia and diarrhoeal diseases, contribute significantly to child mortality, especially in poor and remote areas (Sarfo et al., 2023; World Health Organization, 2020). This is mostly due to health system bottlenecks that affect effective child healthcare service delivery. Health system bottlenecks are impediments to the provision of healthcare services that meet the expectations of various stakeholders (Tanahashi, 1978). Specifically, this study focuses on healthcare infrastructure and medical supply constraints that serve as bottlenecks to the delivery of child healthcare services in a rural setting. The shortage of anti‐malaria medications for instance, made caregivers to resort self‐medication and its undesirable consequences (Ameh et al., 2015).

The implementation of globally effective strategies, such as the Integrated Management of Neonatal and Childhood Illness (IMNCI), has contributed significantly to reducing the impact of malaria, pneumonia, malnutrition and diarrhoeal diseases and improving child health outcomes (World Health Organization, 2014). IMNCI is an integration of the different approaches to tackle the main causes of child mortalities in poor countries. A key strategy component is a consistent supply of adequate essential basic healthcare resources at the primary healthcare level to achieve desired results (Febir et al., 2015). Effective child healthcare services require an adequate supply of essential material resources and infrastructure for health facilities to be able to provide access to effective services to manage sick children timeously (Anarwat et al., 2021; MICS, 2017; Watson et al., 2018). The improvement rate in under‐5 mortalities in Ghana has been quite slow, with under‐5 mortality currently recorded 40 per 1000 live births (Ghana Statistical Service & ICF, 2023). Evidently, prompt and effective child healthcare services contribute to reducing child morbidities and mortalities (Anarwat et al., 2021; Okereke et al., 2015; Watson et al., 2018; Zhao et al., 2021). Unfortunately, in Ghana, over half of health facilities at the primary level lack basic infrastructure and equipment, including basic medical supplies to effectively deliver healthcare services to the populace (Ministry of Health, 2020).

The Sustainable Development Goal (SDG) 3 encourages all countries to work towards attaining Universal Health Coverage (UHC) by ensuring access to quality essential health services by all, including children (World Health Organization, 2018). It is therefore important to explore health system bottlenecks encountered in the delivery of quality child healthcare services so that appropriate interventions can be put in place to address them. However, there is a dearth of literature exploring the combined experiences of service providers and users about the bottlenecks in rural Ghana. The aim of this study is to describe the experiences of nurses and caregivers regarding the health system bottlenecks that impede the delivery of child healthcare services in a rural district in Ghana.

## 2 MATERIALS AND METHODS

### 2.1 Design

A qualitative study was conducted using an exploratory, descriptive design to gain insight into the experiences of nurses and caregivers of children under‐5 regarding the health system bottlenecks that impede the delivery of safe quality child healthcare services in a rural setting.

### 2.2 Setting

We conducted this study in health facilities and communities within the Nkwanta South Municipality, the largest district in the Oti Region in Ghana. This is largely a rural district, with most of the communities located about 10–40 km (3–25 miles) from the capital. The highest level of healthcare delivery at the primary level is the district hospital, with Community‐based Health Planning and Services (CHPS) compounds being the lowest (Ministry of Health, 2016). The CHPS compounds serve to bridge access gaps in healthcare delivery, especially in rural areas. Data were gathered in eight communities, two hospitals, one health centre and three CHPS compounds. The selection of these facilities reflected the different locations and levels of healthcare delivery within the municipality.

### 2.3 Population and sampling

The study population consisted of three different groups: nurses directly involved in the provision of child healthcare services, caregivers of children under 5 years of age who utilised child healthcare services and caregivers of children under 5 years of age who did not utilise the services. Both the nurses and caregivers were purposively selected based on predefined eligibility criteria. Nurses who worked in public health facilities within the municipality and had been directly involved in the provision of child healthcare services for at least the past 6 months were included in the study. The eligibility criteria for the caregivers who utilised the available child healthcare services were that they should be taking care of a child less than 5 years of age. They attended any of the public health facilities to access child healthcare services at least twice within the past year. On the other hand, those caregivers who had not used the available child healthcare services within the past year, even though there was a need to do so, were included as non‐user caregivers. The sample of 26 participants consisted of 10 nurses, nine caregivers who utilised the available child healthcare services and seven caregivers who did not.

### 2.4 Data collection

All the nurses and six of the caregivers who utilised the available child healthcare services were recruited at health facilities and the rest from communities. Before data collection commenced in the health facilities, the nurse managers introduced the field researcher to the nurses and caregivers recruited for the study. Also, Community Health Volunteers in the PHC clinics linked the field investigator to caregivers in communities. The field investigator approached and invited these caregivers to participate in the study. Before the interview sessions, the objectives of the study were explained to each participant. The participants could choose a suitable interview date and time to minimise disruptions to their daily activities.

Individual, face‐to‐face, semi‐structured interviews were conducted with the participants from January to March 2019. The interviews were conducted by the field investigator (FKN), a doctoral nursing student at the time. There was no predetermined relationship between the interviewer and the participants. Interviews conducted with the nurses and four of the caregivers were in the English language. Interviews with the rest of the participants were conducted in the *Twi* language, a common local language. The interviews were recorded using a digital audio recording device and were played back to the participants for clarification where and when necessary. The recordings were transferred to a personal laptop for storage and processing. Data saturation was attained after 26 individual interviews (Brinkmann & Kvale, 2015; Gray et al., 2017). Field notes and reflections were made by the field investigator before and after each interview and were incorporated into the interview transcripts before data analysis commenced.

### 2.5 Data analysis

The collection and analysis of data were done concurrently, applying the steps outlined in Creswell (2014). Those interviews conducted in English language were transcribed verbatim by two trained research assistants. The entire interviews conducted in Twi were transcribed and translated into English by a language expert. The transcriptions were done immediately after each interview. The coding and organisation of the data were done by the field investigator and an independent coder, applying the steps of coding described by Tesch (1990). The process involved engagement with the raw data to gain preliminary understanding by repeatedly listening to the audio recordings; memos were made at the initial stage. Each interview transcript was then carefully read, and common topics were noted down as codes. These codes were appropriately denoted using appropriate words and phrases. Similar codes were clustered and formed into columns. Categories and themes were identified from the columns and matched with their appropriate descriptive topics.

### 2.6 Ethics

Ethical approval for the study was granted by REDACTED and REDACTED. Written permissions were obtained from the Regional Health Directorate, the Municipal Health Directorate and the Municipal Assembly prior to data collection. Additional permission was obtained from the management of the hospitals involved in the study. Prior to the data collection, written informed consent was obtained from each participant. The contents of the participant information sheet were verbally translated into *Twi* for those participants who could neither read nor comprehend the English language; this was done in the presence of a witness, who also signed the consent form to indicate that the translation and all clarifications were done accurately. To uphold the participants' right to privacy and confidentiality, the interviews were held at locations agreed upon with the participants, and their responses were made anonymous.

### 2.7 Rigour

We applied the criteria of credibility, dependability, transferability and confirmability as outlined by Lincoln and Guba (cited in Polit & Beck, 2017) to ensure the trustworthiness of our study. During the data collection process, we ensured credibility by conscientiously following the interview guide; and kept to the subject matter of the interview as part of reflexivity.

Also, we collected data from both caregivers and nurses to capture the divergent perspectives of physical and financial access barriers to child healthcare service utilisation. Furthermore, an independent coder was engaged in the coding process, which added to the credibility of the study. To uphold transferability, a thorough description of the entire research process, including an in‐depth description of the setting, context, data collection and analysis processes, has been included so that other researchers and readers could replicate the study in similar settings or with similar participants. We have also clearly defined the inclusion and exclusion criteria for all participants, thus ensuring that only participants who qualified by way of experience and location were recruited for the study. To ensure confirmability, we maintained a neutral point of view in the data analysis so that participants' perspectives were adequately captured, as evidenced by the inclusion of direct quotes from participants.

Engaging an independent coder and audit trail ensured the dependability of the study.

## 3 RESULTS

### 3.1 Participants' characteristics

The demographic characteristics of the nurse and caregiver participants are shown in Table 1 and Table 2, respectively. The sample size of 26 participants comprised 10 nurses, 9 of the caregivers utilised the available child healthcare services and seven who did not. The age of the nurse participants ranged from 27 to 35 years with 6 being females. Six of caregivers had no formal education; 5 attained or dropped out of basic education; 4 completed senior high school; and 1 had attained a tertiary education. Farming constituted the predominant occupation of the caregiver participants as the majority (10) were engaged in peasant farming; 2 were unemployed, while the remaining 4 were made up of a teacher, a baker, a hairdresser and a healthcare volunteer. While 10 of the caregiver participants were married, 1 was divorced, 1 was widowed and 2 were co‐habiting with the father of their youngest child. In terms of religion, 10 out of the 16 caregivers were Christians, 2 were Muslims, and the remaining 4 were traditionalists. All the caregiver participants were female. The ages of the youngest child of each caregiver ranged from 3 months to 4 years, with most of the children being between 2 and 4 years old.

**TABLE 1:** Demographic characteristics of nurse participants.

Participant label	Age	Sex	Rank	Work experience (years)
Hosp. nurse 1	31	Female	CHN	6
Hosp. nurse 2	28	Male	EN	2
Hosp. nurse 3	35	Female	EN	3
Hosp. nurse 4	31	Male	SNO	6
Hosp. nurse 5	27	Male	SN	3
Hosp. nurse 6	33	Female	NO	5
Hosp. nurse 7	30	Female	SSM	5
PHC Nurse 1	33	Female	SCHN	6
PHC Nurse 2	27	Male	CHN	2½
PHC Nurse 3	33	Female	RCN	6

Abbreviations: CHN, Community Health Nurse; EN, Enrolled Nurse; NO, Nursing Officer; PHC, Primary Health Care; RCN, Registered Community Health Nurse; SCHN, Senior Community Health Nurse; SN, Staff Nurse; SNO, Senior Nursing Officer; SSM, Senior Staff Midwife.

**TABLE 2:** Demographic characteristics of caregiver participants.

Participant label	Age	Level of education	Occupation	Marital status	Religion	Parity	Last child's age (months)
Caregiver 1	60	None	Farming	Widowed	Christianity	11	48
Caregiver 2	23	Secondary	Farming	Co‐habitation	Christianity	1	12
Caregiver 3	33	Basic	Hairdressing	Married	Christianity	3	30
Caregiver 4	23	Secondary	Farming	Married	Christianity	1	30
Caregiver 5	26	Secondary	Unemployed	Single	Christianity	1	18
Caregiver 6	27	None	Farming	Married	Islam	4	24
Caregiver 7	27	Tertiary	Teaching	Married	Christianity	1	9
Caregiver 8	40	Secondary	Healthcare volunteer	Co‐habitation	Christianity	3	24
Caregiver 9	32	None	Baker	Married	Traditional	3	12
Non‐user 1	45	Basic	Farming	Single	Christianity	4	48
Non‐user 2	47	None	Farming	Married	Christianity	8	36
Non‐user 3	35	None	Farming	Married	Traditional	6	7
Non‐user 4	29	Basic	Farming	Married	Islam	3	24
Non‐user 5	40	None	Farming	Married	Traditional	2	24
Non‐user 6	31	Basic	Unemployed	Divorced	Christianity	2	3
On‐user	37	None	Farming	Married	Traditional	5	3

Two main themes emerged from the semi‐structured interviews with the participants as depicted in Table 3.

**TABLE 3:** Themes and sub‐themes that emerged from the data analysis.

Theme	Sub‐themes
1. Inadequate in‐patient infrastructure	1.1 Lack of space 1.2 Poor hygiene facilities 1.3 Exposure to theft and intrusion
2. Inadequate medical supplies	2.1 Inadequate and outmoded equipment 2.2 Shortage of essential consumables 2.3 Shortage of medication 2.4 Effect of shortage of child healthcare

## 4 THEME ONE: POOR STATE OF IN‐PATIENT INFRASTRUCTURE

Both nurses and caregivers (users and non‐users) exposed the poor state of infrastructure in the facilities that provided in‐patient services to sick children, including the lack of space, stranded caregivers, poor hygiene facilities and security concerns.

## 5 LACK OF SPACE

Nurse participants described the situation where they admitted patients beyond the capacity of the children's wards in their hospitals. There are only two hospitals in the Nkwanta South Municipality with the capacity to admit and manage sick children. This situation results in caring for sick children on the floor because all the beds are occupied.

our beds are not enough; the wards are not spacious too (Hosp. Nurse 1:).

sometimes admissions become so much that we even have to keep some of the children on the floor because the beds are full (Hosp. Nurse 4).

In one of the hospitals, the lack of adequate space made the segregation of sick children according to age and/or severity of their conditions impossible, increasing the risk for cross‐ infections, especially for young infants with inadequately developed immunity.

we don't have a NICU, for now we use part of maternity for NICU, so you have no choice but to mix them [neonates] with those cases that we know very well that they might end up getting some of the infection (Hosp. Nurse 6).

Providing nursing care to children who are lying on the floor posed physical challenges to the nursing staff, including musculoskeletal disorders emanating from them having to adopt awkward postures when providing care to patients in this context. Additionally, the use of certain equipment such as infusions on these patients was impossible

Even you the nurse that is nursing the patient on the floor finds it difficult to work. Squatting and giving medication, is difficult. Using the drip stand for the floor patient is not feasible. we don't have drip stands for all the beds, so those on the floor, it becomes difficult to administer IV fluids (Hosp. Nurse 3).

The nurses also found it emotionally upsetting caring for children on the bare floor of the hospital. They described that they felt distressed seeing children on the floor because of a shortage of beds.

(Smiles) …, at times I feel bad because, a patient comes to the hospital and is on the floor instead of being on a bed for treatment (Hosp. Nurse 3).

To reduce the incidence of children having to lie on the floor, nurses described that they added cots in spaces meant for other activities on the ward, resulting in congestion, leaving very little space for the nurses to keep essential items nor space for the children to play while on admission.

Because of the number of beds, which is thirty‐one, we don't have anywhere to improvise and keep [essential items] (Hosp. Nurse 4).

This is our playroom (pointing to a room labelled as ‘resuscitation room’) unfortunately, it is now a ward. It was supposed to be a creche (…) (Hosp. Nurse 5).

According to them, there was no arrangement for the parents of children on admission to have a befitting place to spend the night.

On other wards, they have a sleeping place for the relatives but here [children's ward] they don't have (caregiver 2).

The caregivers described as uncomfortable when they are compelled to share the small available space with their sick children, irrespective of the child's age.

Whether it is a baby or a grown up the place is not big enough for you to sleep with the person (caregiver 5).

the beds are very small (…)I was just managing like that. The mattress is very small, and you will not be happy if you sleep and your baby cannot sleep so I was just struggling till the day I was discharged (caregiver 7).

Most caregivers resorted to unconventional measures to muddle through these challenges. According to them, they slept at unorthodox places within the precincts of the wards.

If you have a cloth, then you use it. For me, so I usually pack these stools here and lie on them (caregiver 2).

For the mothers many at times we sleep on the floor because the place is not big enough to sleep beside the person you've sent to the hospital. Even if you sleep together the person will not be comfortable so you have to lay either your mat or your cloth on the floor then you sleep (caregiver 5).

Caregiver participants expressed their unhappiness about this situation. Whereas some of the caregiver participants expressed their helplessness under the circumstances, others were concerned about the health implications of sleeping on the bare floor.

Doctors advised that we shouldn't be sleeping on the floor and we will get a sickness called pneumonia. And at the hospital too that we sleep on the floor, so I don't know how they think about us (caregiver 5).

## 6 POOR HYGIENE FACILITIES

The caregiver participants further expressed disquiet about the poor state of the facilities for the maintenance of personal hygiene. According to the participants, both patients and relatives shared the same toilet facilities, which were in a bad state.

The toilet facility is outside. It is not clean. They go to toilet around the whole place like that. Even how to enter [the toilet facility] is a problem (caregiver 2).

They described the place as congested, nasty and always messed up with no running water to flush after use. Besides, the place is not cleaned regularly by the caretakers, thus making it uncomfortable to use these facilities.

They have a bath house, but they are not keeping the place neat. There are a lot of people bathing there but the woman who is supposed to be cleaning the place doesn't do anything about it. The bath house is very dirty so when I went there, I was not happy about how the place was (caregiver 7).

Some people will go there and bath and remove their pads, you will see polythene bags, dirty rags all kinds of things in the bath house, they are no more cleaning the place again (caregiver 5).

Again, the caregiver expressed unhappiness and discomfort about the lack of privacy in the washrooms. They also expressed the fear that the bathroom could serve as grounds for infection transmission.

You can be in the same bathhouse with about two, three and even four people at the same time (caregiver 4).

It didn't feel comfortable because I feel like every child who has bathed there has their own sickness and then I would also bring my child there to bath. And for the children too, they will just sit on the floor there whiles you are about to bath him. (caregiver 8).

To deal with the challenges of poor hygiene facilities, some of the caregivers went outside the hospital to maintain their personal hygiene, and other participants sneaked their sick children out of the wards to bathe them at home. For other participants, they dressed the children in diapers so that they could ease themselves into the diapers.

You see that they (children) are not matured, so we usually, dress them with pampers. If they can talk, you see that they will tell you that they want to go to toilet, then you send them outside. You take them to the adult place outside (caregiver 2).

Because we have a house at Nkwanta, I usually go there to bath and come. (caregiver 2).

Because my house is not far from here, I carry her home and bath her, then we return to the ward. We don't inform the nurses that we are going home to bath. (caregiver 1).

## 7 EXPOSURE TO THEFT AND INTRUSION

According to the participants, the ward environment was fraught with safety challenges, which exposed the nurses and the patients to thefts and intrusion by unauthorised persons. The participants described feeling insecure as there were no security personnel stationed at the wards to ensure safety. The ward environment, including the human and material resources, was thus exposed to thefts and physical harm.

…the unit does not have security on its own. There are some facilities that have somebody sitting at the gate and restricting visitors and checking other things, we don't have something like that (here) (Hosp. Nurse 4).

Having to maintain their own security while providing care to sick children often distracted the nurses and affected the quality of nursing care provided to the sick children.

Even when you are concentrating on a case at the nurses' station, we don't know what is going on, on the ward or the other side (Hosp. Nurse 1).

The nurse participants described the difficulties they encountered when trying to enforce the facilities' protocols on visiting hours. Also, caregivers easily absconded with their children on the blind side of the nurses.

If they (visitors) come at the wrong time, that is what I am saying, we are forcing ourselves to stop them, because we don't have the security personnel (Hosp. Nurse 1).

That is why we have even moved our table to the corridor so that we can monitor but still they (patients) end up absconding. Some abscond and don't even come back again (Hosp. Nurse 4).

## 8 THEME TWO: INADEQUATE MEDICAL SUPPLIES

Nurses in both hospital and clinic settings experienced a persistent lack of essential equipment and consumables for the delivery of child healthcare services. The shortage made nursing care difficult and negatively affected the experiences of caregivers of sick children who come to sick child healthcare services.

## 9 INADEQUATE AND OUTMODED EQUIPMENT

The nurse participants described their experiences regarding the lack of basic equipment for the delivery of child healthcare services. The equipment included beds/cots, incubators, patient monitoring instruments and essential medicines.

Nurse participants expressed that the beds and cots in the children's wards were non‐ adjustable and dilapidated making it difficult to nurse children on them.

The beds are not adjustable so even if you want to elevate the head or you want to elevate the foot‐end, you have to find some box or something and put under the mattress (Hosp. Nurse 4).

Even beds, some of them cannot be lifted. (…)And some of the cots too, you can't lift the head up (Hosp. Nurse 5).

Participants described that these beds endangered the safety and well‐being of children who are placed on them due to the lack of side‐rails and restraints resulting in falls.

If you look at our incidence book, patients falling from the bed is one of the major incidents we record here, because of the lack of side rails for the bigger beds (Hosp. Nurse 4).

Children are vulnerable and require adequate protection to ensure their safety and well‐ being. Particularly, sick children are at increased risk of sustaining injuries and endangering their lives if not adequately protected.

They also explained that the facilities lacked basic equipment necessary for child healthcare delivery.

No, we don't have any specific equipment for the management of paediatric case. (PHC nurse 3).

we don't have cardiac monitors or anything to do the monitoring. We only use the normal digital thermometers, the manual pulse oximeter. We don't have a fixed monitor (Hosp. Nurse 4).

The nurses described that they improvised some of this essential equipment to provide care for sick children.

We don't have any incubators. But we have made some cots with radiant warmers. Specifically, we use bulbs to generate heat. That's where we manage them (neonates) (Hosp. Nurse 7).

The perennial shortage of essential equipment means that the few available ones are constantly overused, leading to frequent breakdowns. A situation which made the delivery of timely patient care more difficult.

Because we use them frequently and it is not enough, the battery goes low quite often, or the gadget doesn't work at all;… As I talk now, we have about four digital thermometers to run four hourly temperature checks for thirty‐one patients. Within a few months, it gets spoilt. It makes the work so difficult (Hosp. Nurse 4).

Our suction machines, concentrators; sometimes we'll have more than two babies on oxygen. For some, you may need to use the (oxygen) concentrator and it's spoilt because of the pressure (Hosp. Nurse 7).

Some caregiver participants expressed worry over the use of some of this equipment for several children without proper cleaning. The caregivers were worried that this practice could lead to the transmission of infections.

It is the checking of the temperature that they use the same thing (thermometer) for all the children. Hmmmmm, … (long pause). I don't know whether that can transfer diseases to the children. But, for me I think there should be something that if they use it to check one child, they should clean it before using it on another child. (caregiver 2).

## 10 SHORTAGE OF ESSENTIAL CONSUMABLES

The nurses experienced a persistent shortage of basic consumables, which are non‐durable items that are mostly meant to be disposed after use. These consumables items such as patient folders, medication trays and oxygen and bag valve masks. The situation often caused them to improvise with whatever was available to them. They explained that the improvisation affected the smooth delivery of child healthcare services as they were sometimes left helpless.

We don't have folders. So, we have an exercise book that when they(children) come, we write the data with their OPD numbers on it (PHC Nurse 2).

… for instance, when you want to give medication to a child you are supposed to have everything set. You are supposed to have a folder, …but over here we have most of our things being a leaflet, so we have to collect these things bring them together and clip it; what if the clip used to hold it is removed? (Hosp. Nurse 1).

The shortage of these logistics rendered the nurses ineffective and contributed to patient dissatisfaction with healthcare delivery.

Sometimes you come for duty at night, a baby is gasping, or the oxygen saturation is low, and you check your cylinder, and there is no oxygen, you have to run to the other wards in search of oxygen, if they don't have then you are doomed (Hosp. Nurse 1).

The nurse participants further described some of the available equipment as unsuitable for use in children. These items were mostly meant for use in the adult units; however, the nurses described that they had to improvise them for use in the children's units.

And we don't have some few things like ambu‐bag; especially the paediatric size of masks. We just have a few things that we use to improvise for the emergency tray (Hosp. Nurse 4).

Just as I said, the age‐appropriateness (of the equipment) is a factor. They are almost adult items we improvise. So, we don't actually have specific paediatric items, just that we are able to manage with the adult ones (Hosp. Nurse 6).

Narrations from the nurse participants revealed that monitoring patients, which is an essential nursing activity, was not performed as required because of equipment and logistical challenges. The quality care rendered to sick children could not therefore be guaranteed.

Nurse managers could, however, not hold nurses accountable for the care they provide to sick children because the basic logistics were not available to ensure quality nursing care.

So, if we have to monitor a case that requires monitoring every fifteen or thirty minutes, it becomes very difficult, and staff don't do it. Honestly speaking, because we don't have the fixed monitors, we are not able to do the strict monitoring of the cases we have to monitor closely enough… Sometimes, I don't really fault them (nursing staff) so much. Because the things they require for monitoring are not there (Hosp. Nurse 4).

The nurse participants further explained that they are compelled to reuse some of the items that were meant for single use. This risky practice could endanger the life and health of sick children, particularly pre‐term neonates with inadequate immunity.

And some of the things that are supposed to be disposable we keep reusing them; we decontaminate and re‐use just because we don't have enough. Like these nasal prongs (pointing to disposable nasal prongs), they are disposable, but you need a very small one for a preterm. You have only one so after using it, instead of throwing it away, you decontaminate, wash it and put down for re‐use (Hosp. Nurse 7)
.

In other instances, the nurses improvised with available resources to care for sick children. They usually used commonly available materials to enable them to continue to render care to sick children.

The items, for instance when you are setting the cannula, any IV infusion, we suffer with some of the items, eerhm, the giving sets, the tourniquet, we use this rubber tourniquet (shows a giving set that has been cut to improvise as a tourniquet), which is not advisable (Hosp. Nurse 1).

We don't have a well‐structured emergency tray. So, we just improvise some wooden tray (Hosp. Nurse 4).

To avert the danger of contaminating and infecting themselves because of the shortage of essential consumables, the nurses used their own resources to purchase some of these items.

Sometimes we do use our own money. Because you have to prevent yourself from some infections. Some [nurses] come together and decide on what to do. We do a little contribution among ourselves and buy the gloves and other essential items from town (PHC Nurse 1).

As they have no place to keep patients' medications, the nurses indicated that left the medication of children with the caregivers. The caregivers, however, out of ignorance, ended up administering the drugs inappropriately, leading to underdosing or overdosing of the children.

so what we do is that we leave the drugs with the patients, even though ideally, it is not supposed to be so. Because the parents we have here are not much educated, so sometimes when the child is having some difficulty, they end up giving excess of the medication. …So, by the time you will go and administer, the child had already taken excess and you may not know (Hosp. Nurse 4).

Being aware of the dangers of this practice, the nurses expressed the worry that the children could even consume these medications inappropriately out of curiosity.

Because they are kids; they are adventurous. Every day, a child wants to experiment with something, so personally, I feel that with kids, their medications are not supposed to be with them. (Hosp. Nurse 6).

## 11 SHORTAGE OF ESSENTIAL MEDICINES

The nurses described the persistent shortage of essential medicines as a common occurrence. According to the participants in the PHC clinics who got their supplies of medical products from the regional medical stores, these supplies mostly lasted just a few days.

Almost every month. For a quarter, we will have medicines for one and half months and for the remaining two months, we done have any medicine (PHC Nurse 1).

Every two months, we run out of stock on drugs especially ‘Para’. Because we use it in almost every case. That usually finishes earlier than the other medicines (PHC Nurse 3).

These narratives were corroborated by the caregiver participants. According to the caregivers, they were mostly given prescriptions to buy these medications from drug stores.

The challenge they (clinics) face most is that they don't get drugs. If you come here, they will treat you very nicely but they won't get the medicines so they will write for you to go and buy (caregiver 7).

But what Kechebi is lacking is, at times when you come, they write the medicine for you to go and buy in drug stores… If you ask, they will say they are lacking the drugs (caregiver 5).

The persistent shortage of essential medicines at the PHC facilities dissuaded caregivers from utilising the available child healthcare services.

Anytime I go to the hospital (PHC clinic), I will be given a prescription to go and buy medication from outside the health facility. There has not been a time that I have ever been given medication from the health facility. that is why I stopped [attending that clinic] (non‐user 4).

Participants in the hospitals similarly experienced periodic shortages of essential medicines for child healthcare. In such instances, the caregivers are made to buy these medications from outside the hospitals.

In case of emergency if we need a particular drug, we write for the patient to buy. When there is none at the stores then they go and buy from town (Hosp. Nurse 3)

After they were done attending to my child, the said the medication they are supposed to give him was not available so, they wrote it for me to go and buy. (caregiver 9).

The shortage of these essential medicines put the nurses who care for children in distress. One nurse participant explained the psychological distress nurses go through, knowing that the medication they were administering would not provide the relief that the child required.

…when you come to work and see a client in pain and you are administering paracetamol and brufen (ibuprofen) and deep down your heart, you know that if I were in this pain these things will not solve [it] and see them crying and they are kids that cannot really express themselves then you are hurting. You are not the one in pain but psychologically, you are suffering the pain with your patients (Hosp. Nurse 6).

The nurse participants blamed the persistent shortage of medication on the regional medical stores, which supply these medicines to public facilities within the regions. The participants explained that they are usually supplied only a percentage of their requisition for each quarter. These medicines get used up within a very short time, leaving them with nothing to offer their clients.

Now they say we should request from the regional stores. … Maybe you ask for 100 bottles of paracetamol syrup, they will only give you 25. You can even use all the 25 in a day. And you are supposed to request for drugs every three months, so when you finish your consignment in a day or a week, it means you have to wait for another three months, before you make a new request (PHC Nurse 1).

To deal with this in the PHC clinics, the nurses purchased medications from private suppliers to sell to caregivers. This practice, however, infuriated some caregivers, especially those with insurance.

When they come with their children, we tell them we used our moneys to buy the drugs so if they (caregivers) have money we will sell it to them if they don't have, we will write it for them to go and buy (PHC Nurse 2).

But about 40% [of caregivers] too are like ‘why don't you have it?’, ‘why should I go and buy when I have insurance?’ and insurance promised us free health care so why do I have to take money and buy medicine? And remaining percentage too will tell you they don't have money (PHC Nurse 3)

## 12 EFFECT OF MEDICATION SHORTAGE ON CHILD HEALTHCARE

The nurses lamented that the shortage of essential medication made them ineffective as the gatekeepers of the healthcare system. According to them, the situation was counterproductive to effective child healthcare delivery as some of these drugs were required daily.

I will say that for now, the only challenge hindering our service provision is the medication. Even, sometimes, I say that when you are a nurse and there is not medication or things that you need to work with, you are ‘useless’. Because you will be sitting down doing nothing, people will come, and you tell them there is not

medication (PHC Nurse 1)

So, you'll see a child being brought in who has already convulsed in the house, but the ‘magic’ is not there. These para suppositories are not there to at least stabilise the condition (PHC Nurse 3).

The participants asserted that caregivers only visited PHC clinics with mild conditions. They side‐stepped the gatekeepers and went straight to the hospitals when they perceived their children's situation as severe.

Sometimes, the people by‐pass us because of shortage of drugs and go to level C (district hospital). … When they come and there is no drug and we tell them to go to the next level, then the news spreads (PHC Nurse 1).

So normally, if your child's sickness is worse, you don't go there (PHC clinic), unless you have a child who is suffering from ‘teething’; that is when you go to Keri [CHPS compound] (caregiver 2)

Some caregivers resorted to self‐medication instead of visiting PHC clinics where they will not be given medication for their sick children.

so many at times people like to go to the pharmacy than to come to Kechebi health centre, for instance. They have been saying that when you come to the clinic over here you won't get any medicine (caregiver 5).

## 13 DISCUSSION

In this study, we explored the experiences of nurses and caregivers about the health system bottlenecks that impede the delivery of child healthcare services timeously in a rural district in Ghana. Two themes emerged from the analysis of semi‐structured interviews we conducted with the participants.

The health system bottlenecks were encountered both in in‐patient and out‐patient child healthcare services settings. The deplorable state of in‐patient facilities was mostly in the form of congestion in the wards, no arrangement for caregivers' accommodation, poor hygiene facilities and exposure of health workers and patients to theft and intruders. The limited in‐patient space resulted from the increased number of admissions of sick children, as reflected in the persistent high bed occupancy and the resultant caring for sick children on the floor. These findings are consistent with previous studies regarding the poor state of in‐ patient child healthcare service facilities in resource‐constrained settings. Similar studies in the West Africa sub‐region have found that the quality of maternal and child healthcare services is low (Koulidiati et al., 2018; Shibanuma et al., 2018). Nursing children of different ages and with different conditions in the same space could contribute to cross‐infection, placing neonates at greater risk of acquiring nosocomial infections. These findings are consistent with those of Kizza et al. (2011), who determined that overcrowding negatively affected the quality patient care in Uganda. A study by Dalinjong et al. (2018) found that healthcare facilities in rural Northern Ghana admitted and managed patients on the bare floor due to insufficient beds and limited space. The lack of privacy hampered the dissemination of information to caregivers, as negative information caused distress among other caregivers (Kilcullen & Ireland, 2017). Furthermore, increased work demands, and inadequate resources have been noted to contribute to burnout among nurses. The poor state of in‐patient facilities negatively impacted on the physical and psychological well‐being of nurses who encountered these challenges. The current state of in‐patient care could have a less desirable impact on the experiences of caregivers and their children and their decision to utilise child healthcare services in the future (Agunwa et al., 2017; Anichukwu & Asamoah, 2019).

The delivery of quality child healthcare services relies heavily on availability of medical supplies including, logistics and modern gadgets which are child friendly. The study also discovered the lack of basic equipment for the delivery of child healthcare services.

Additionally, the available equipment were mostly overused, leading to frequent breakdowns. Previous studies made similar findings in resource‐constrained settings (Febir et al., 2015; Koulidiati et al., 2018). Additionally, dilapidated and outmoded beds in the wards aggravated the situation making nursing care difficult. The nurses were therefore compelled to adopt unconventional approaches to manage the situation, including failing to monitor patients as required, referral of critical cases to other facilities or using their own resources to purchase basic equipment to facilitate work. All these perceived mitigating measures, in the long run, could contribute to poor patient outcomes.

Equally important in child healthcare is the consistent availability of quality medicines for the urgent management of sick children. The delivery of child healthcare in the study setting was characterised by the persistent shortage of essential and basic life‐saving medications.

The shortage was experienced both in the PHC clinics and hospitals. Studies have shown that a persistent shortage of essential medicines affects the delivery of child healthcare services, especially in PHC settings (Kinney et al., 2010; Ntambue et al., 2016). Several other studies have noted that poorly equipped healthcare facilities, which render sub‐standard care to clients, were noted as a challenge to service provision and utilisation in most developing countries (Abraha et al., 2019; George et al., 2018, p. 6; Kabia et al., 2019).

The study explored in detail the perspectives of multiple stakeholders in different settings regarding the health system bottlenecks that affected the delivery of child healthcare services in a resource‐constrained setting. The study advanced the discourse in this regard. However, the study has some limitations. First, the perspectives of other stakeholders, including community and opinion leaders, and healthcare managers, were not explored. Second, the qualitative nature of the study could not permit the quantification of the influence of these bottlenecks on the overall deplorable state of child health in rural areas. Future studies should consider using quantitative methods to assess the extent of influence and also including the perspectives of other major stakeholders.

## 14 CONCLUSION

The health system bottlenecks are a setback to the delivery of quality child healthcare services timeously. This has the tendency to affect the treatment and hospitalisation outcomes and eventually impact the state of child healthcare negatively. To achieve Universal Health Coverage requires concerted efforts to remove all barriers to accessing and utilising healthcare services. So, if the facilities are to meet the expectations of caregivers and reduce child mortalities, then adequate measures must be implemented to address these bottlenecks.

It is recommended that the PHC clinics should be provided with the required medical supplies so that they can attend to sick children in the communities and reduce the rate at which caregivers' troupe to the hospitals. Also, the local authorities should work together to increase essential medical supplies for child healthcare delivery to facilities, especially in rural areas where caregivers have limited options. Also, adequate training should be provided to healthcare workers so that they can properly maintain this equipment to prolong their lifespans. Training in logistics management and stockkeeping could also go a long way to reduce erratic shortages of essential supplies in the health facilities.

## AUTHOR CONTRIBUTIONS

FKN conceptualised the study and conducted the field interviews, performed the data analysis and developed the first draft of the manuscript. Guidance for the study design was provided by ER, SJB and MW. All authors reviewed and approved the contents of the manuscript. The methodological content and alignment of the manuscript was provided by ER. All authors approved the final version of the manuscript, prior to submission.

## FUNDING INFORMATION

The doctoral study from which this data set was drawn received funding from the Postgraduate Research Scholarship fund of the Nelson Mandela University.

## CONFLICT OF INTEREST STATEMENT

The authors declare that they have no conflict of interests that could have appeared to influence the data contained in this manuscript.

## RESEARCH ETHICS COMMITTEE APPROVAL AND CONSENT TO PARTICIPATE

Ethical approval for the study was granted by the Nelson Mandela University Research Ethics Committee (reference number: H18‐HEA‐NUR‐018) and the Ghana Health Service Research Ethics Review Committee (reference number: GHS‐ERC014/11/18). All methods were carried out in accordance with relevant guidelines and regulations. Prior to participation, each participant signed the informed consent form.

## PARTICIPANT CONSENT STATEMENT

All participants granted permission for excerpts of verbatim quotations from participant interviews to be included in any publication stemming from the study.

## Data Availability

The data sets for the study are not publicly available; this is due to the study's ethical requirements to ensure respondent anonymity in reporting, and confidentiality in participating in the study. These data sets are, however, available from the corresponding author, upon reasonable request.
